# Lipid-based nano-delivery systems for skin delivery of drugs and bioactives

**DOI:** 10.3389/fphar.2015.00219

**Published:** 2015-09-30

**Authors:** Susan Hua

**Affiliations:** The School of Biomedical Sciences and Pharmacy, The University of NewcastleCallaghan, NSW, Australia

**Keywords:** lipid-based nano-delivery system, liposomes, niosomes, Transfersomes, ethosomes, dermal drug delivery, transdermal drug delivery

## Introduction

Topical drug delivery across the skin can offer many advantages, such as confer sustained drug release, lower fluctuations in plasma drug levels, circumvent first-pass metabolism, improve patient compliance, and provide local (dermal), or systemic (transdermal) effects (Schäfer-Korting et al., 2007; El Maghraby et al., 2008). However, the barrier function of the skin, exerted by the horny layer of the stratum corneum, impairs the penetration and absorption of drugs (Bouwstra and Ponec, 2006). This layer prevents the penetration of hydrophilic compounds much more efficiently as compared to lipophilic compounds (Bouwstra et al., 2003; Bouwstra and Ponec, 2006). Therefore, there has been wide interest in exploring new techniques to increase drug absorption through the skin. Novel topical drug delivery systems, with the use of nanotechnology in dosage form design, have been used to facilitate overcoming the skin barrier. This article will summarize recent findings of lipid-based nano-delivery systems for skin delivery of drugs and bioactives agents.

## Skin barrier and permeability

The main barrier of the skin is located in the outermost layer, the stratum corneum. The stratum corneum consists of corneocytes surrounded by lipid regions. As most drugs applied onto the skin permeate along the lipid domains, the organization and composition of the lipid component is considered to be very important for the skin barrier function. This layer generally consists of long chain ceramides, free fatty acids and cholesterol (Bouwstra et al., 2003; Schäfer-Korting et al., 2007). Additional defensive features of the stratum corneum that have a negative influence on skin penetration, include the low pH, the presence of enzymes on the skin, and the transcutaneous concentration gradient (Geusens et al., 2011). The factors that affect the skin absorption of topically applied agents are size, shape, superficial charges, lipophilicity, presence of penetration enhancers, type of formulation, and physical state of the stratum corneum (Verma et al., 2003a).

Skin permeability of topical agents is often assessed using *ex vivo* (human or animal skin) or *in vitro* (e.g., parallel artificial membrane permeability assay—PAMPA) techniques (El Maghraby et al., 2008; Yu et al., 2015). As the majority of studies have generated data from animal models, this has posed a number of issues in relation to significance of the results to humans, as skin permeability differs between species. For example, mouse skin is much thinner and contains more hair follicles—increasing the odds for transdermal delivery (El Maghraby et al., 2008; Geusens et al., 2011). The use of skin PAMPA technology for high-throughput analysis of topical drug permeability has shown promise in assessing passive absorption processes of free drugs (Sinkó et al., 2012). However, the use of artificial membranes for evaluating nano-delivery systems is still limited, with more research required to establish more accurate and consistent results. In general, the different experimental parameters such as formulation, experimental setting, species, gender, and age of skin donors, often make it challenging to compare similar studies (Geusens et al., 2011).

Furthermore, diseased skin is often characterized by a reduce barrier function and an altered lipid composition and organization. In diseased states, often the activity of one or more enzymes involved in the synthesis of the barrier lipids is altered compared to that in normal skin. Percutaneous inflammation can also influence the permeability of topical agents (Bouwstra et al., 2003; Bouwstra and Ponec, 2006). It should be kept in mind that due to the complexity and inter-individual variability associated with different skin conditions, the use of native stratum corneum does not allow detailed systematic studies.

## Interaction of lipid-based nano-delivery systems with the skin

The exact mechanisms by which vesicular systems deliver drugs into intact skin are not yet fully understood. Some proposed mechanisms of action of such vesicles in the modulation of skin permeability have previously been reviewed (Dubey et al., 2007; Elsayed et al., 2007; El Maghraby et al., 2008; Nounou et al., 2008; El Maghraby and Williams, 2009). Four general mechanisms have been reported, which include (i) intact drug-laden vesicle penetration into the different layers of the skin; (ii) lipid vesicles acting as penetration enhancers via their skin lipid-fluidizing property; (iii) direct carrier-skin drug exchange by “collision complex transfer” between the drug intercalated in the lipid bilayer and the surface phase of the stratum corneum; and (iv) lipid vesicle-mediated enhanced transdermal drug delivery via appendageal pathways (e.g., hair follicles and sweat ducts) (Dubey et al., 2007; Elsayed et al., 2007; El Maghraby et al., 2008; Nounou et al., 2008; El Maghraby and Williams, 2009). The transepidermal pathway across intact skin contains two micropathways—the intercellular and transcellular route. As both pathways involve transport through intercellular lipids, research has focused on understanding the composition and organization of these structures in the stratum corneum (El Maghraby et al., 2008).

The mechanism(s) involved is dependent on the formulation, in particular factors such as composition and particle size. For example, particle size of lipid vesicles has a significant influence on delivery of substances into the skin (Verma et al., 2003a,b). Generally, large vesicles with a size ≥600 nm are not able to deliver their contents into deeper layers of the skin. These vesicles tend to stay in or on the stratum corneum and may form a layer of lipid after drying. Vesicles with a size ≤ 300 nm are able to deliver their contents to some extent into the deeper layers of the skin; however those with a size ≤ 70 nm have shown maximum deposition of contents in both viable epidermal and dermal layers (Verma et al., 2003a). Whether these lipid vesicles can pass intact into deeper layers of the skin or not is still a subject of debate (Bouwstra et al., 2003). Nanoparticles below 6–7 nm or 36 nm in size may be absorbed through the lipidic transepidermal routes or aqueous pores, respectively. In contrast, larger agents (10–210 nm) may preferentially penetrate through the transfollicular route (Geusens et al., 2011).

## Lipid-based nano-delivery systems for topical drug delivery

**Figure 1:**
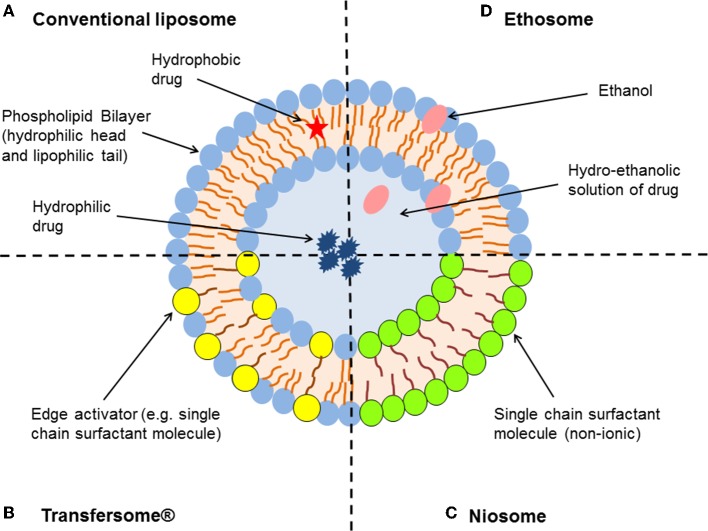
**Schematic representation of the different types of lipid-based vesicular delivery systems**. **(A)** Conventional liposomes generally consist of a lipid bilayer composed of phospholipids and cholesterol, which encloses an aqueous core. Both the lipid bilayer and the aqueous space can incorporate hydrophobic or hydrophilic compounds, respectively (Hua and Wu, 2013). Liposome characteristics can be modified by the addition of surfactants to form **(B)** Transfersomes® and **(C)** niosomes (depending on the ratio of phospholipid to surfactant), or relatively high concentrations of ethanol to form **(D)** ethosomes (Geusens et al., 2011; Vanic, 2014).

Liposomes are well-established nano-sized lipid-vesicles that offer potential value in topical drug delivery. They are formed by one or multiple lipid bilayers that enclose a discrete aqueous phase (Figure 1). Liposomes offer many advantages as drug delivery carriers—for example they are biodegradable, non-toxic, and are able to encapsulate both water-soluble and lipophilic substances (Hua and Wu, 2013; Hua, 2014; Bozzuto and Molinari, 2015). They are similar to the epidermis with respect to their lipid composition, which enables them to penetrate the epidermal barrier to a greater extent compared to other conventional dosage forms. The majority of topically applied liposomes onto the skin will accumulate in the upper layers of the stratum corneum and function more as a “reservoir” providing a more localized action (Kirjavainen et al., 1999; Honeywell-Nguyen and Bouwstra, 2005; Cosco et al., 2008; Geusens et al., 2011).

In order to further enhance skin permeation of encapsulated molecules, alterations in composition and structure of conventional liposomes were made to generate new classes of lipid vesicles with flexible and ultradeformable properties—in particular niosomes, Transfersomes® and ethosomes (Figure 1). Niosomes are non-ionic surfactant vesicles made up of single chain surfactant molecules in combination with cholesterol. These nanoparticles generally resemble the same characteristics as that of liposomes, however are considered more stable (Vora et al., 1998). Niosomes were thought to improve the horny layer properties, both by reducing transepidermal water loss and by increasing smoothness through replenishment of lost skin lipids following fusion to corneocytes (Hofland et al., 1995; Geusens et al., 2011). Niosomes have the ability to modify the structure of the stratum corneum through their surfactant properties, in order to make the layer looser and more permeable (Geusens et al., 2011).

Transfersomes® are considered the first generation of highly elastic or deformable vesicles (Cevc and Blume, 1992, 2001). They are a novel type of liquid-state vesicles that consist of phospholipids and an edge activator, which is often a single chain surfactant (e.g., Sodium cholate, Span 60/65/80, and Tween 20/60/80) that destabilizes the lipid bilayers of the vesicles and increases their deformability by lowering the interfacial tension. This feature is thought to enable Transfersomes® to squeeze themselves through intercellular regions of the stratum corneum under the influence of the transdermal water gradient. They have been reported to penetrate intact skin *in vivo* with an efficiency similar to subcutaneous administration, provided that the elastic vesicles are topically applied in non-occlusive conditions (Cevc and Blume, 1992, 2001; Elsayed et al., 2006, 2007).

Ethosomes are another novel lipid carrier that has shown enhanced skin delivery of encapsulated compounds (Touitou and Ainbinde, 2000; Touitou et al., 2000). The ethosomal system is mainly composed of phospholipids, a relatively high concentration of ethanol (20–50%) and water. Rather than destroying the lipid vesicular structure, Touitou et al. demonstrated that the high alcohol concentration allows the formation of soft, malleable and highly fluid vesicles (Touitou et al., 2000). Ethanol is a well-known permeation enhancer that is suggested to provide a synergistic mechanism with the vesicles and skin lipids (Touitou et al., 2000; Elsayed et al., 2006). The inclusion of ethanol may provide the vesicles with soft flexible characteristics, which allow them to more easily penetrate into deeper layers of the skin. Phospholipid vesicles containing ethanol may also influence the bilayer structure of the stratum corneum to enhance drug penetration (Touitou and Ainbinde, 2000; Elsayed et al., 2006).

Introduction of permeation enhancers into the composition of the newer lipid-based nano-delivery systems was thought to increase both vesicle elasticity and deformability. These vesicles also have the ability modify the stratum corneum to promote impairment to its barrier function, with less well-packed intercellular lipid structure to increase skin partitioning of the drug (Elsayed et al., 2006). These novel vesicles have demonstrated enhanced therapeutic potency compared to conventional liposomes, where deeper levels of skin permeability is required (Elsayed et al., 2007; Geusens et al., 2011).

## Biomedical applications of lipid-based nano-delivery systems

Topical drug delivery is an area of active research as it provides a convenient and effective dosage form for the treatment of local pathological conditions (dermal drug delivery) or as the site for the administration of systemically active drugs (transdermal drug delivery). Lipid-based vesicles have been used to encapsulate a multitude of therapeutic agents, including drug molecules (Hua and Wu, 2013; Bozzuto and Molinari, 2015), gene therapy (Geusens et al., 2011; Monteiro et al., 2014), and bioactive agents (e.g., growth factors and cytokines) (Monteiro et al., 2014). Their use in topical formulations have demonstrated improved treatment of inflammatory skin conditions such as atopic dermatitis, psoriasis and acne; pathological conditions of peripheral tissue such as inflammatory pain, melanoma, and wound healing; and transdermal delivery of systemic agents for vaccinations and conditions such as diabetes (insulin delivery) and hormone replacement therapy (estrogen delivery) (Elsayed et al., 2007; Schäfer-Korting et al., 2007; El Maghraby et al., 2008; Geusens et al., 2011).

Based on recent findings, it has been demonstrated that conventional liposomes are of little or no value as carriers for transdermal drug delivery, as they tend to remain confined to upper layers of the stratum corneum and are not able to penetrate into the granular layers of the epidermis (Kirjavainen et al., 1996, 1999; Honeywell-Nguyen and Bouwstra, 2005; Cosco et al., 2008; Geusens et al., 2011). Conventional liposomes have shown increased effectiveness for local dermal drug delivery across intact skin in comparison to free drug, with the ability to provide prolonged drug release (Hua and Wu, 2013). For example, topical application of loperamide HCl-encapsulated liposomal gel, resulted in potent and prolonged analgesic and anti-inflammatory activity in a rodent model of acute inflammatory pain compared to controls (which did not show any therapeutic effects) (Iwaszkiewicz and Hua, 2014). Topical application of conventional liposomes has also been used for wound healing. It should be noted that the skin barrier properties are compromised with open wounds, which increases penetration and absorption across the skin. For example, topical application of fibroblast growth factor-1 (FGF-1) cDNA encapsulated within cationic liposomes, demonstrated enhanced wound healing to the injured skin of diabetic mice compared to controls (Sun et al., 1997).

The newer classes of lipid vesicles, including deformable lipid vesicles and surfactant-based elastic vesicles, have generated improved *in vivo* and *in vitro* skin delivery of various drugs and bioactive agents for both dermal and transdermal drug delivery (Elsayed et al., 2007; Schäfer-Korting et al., 2007; El Maghraby et al., 2008; Geusens et al., 2011). For example, deformable lipid vesicles have been studied for use as a gene delivery system for topical application. Results have demonstrated enhanced *in vivo* transfection efficiency of plasmid DNA and prolonged retention times, with genes transported into several organs for 6 days once applied on intact skin (Kim et al., 2004). In comparison to deformable lipid vesicles, ethosomes are able to improve skin delivery of drugs under both occlusive and non-occlusive conditions (Elsayed et al., 2006, 2007).

## Future advances

Although the therapeutic advantages of lipid-based vesicles for skin delivery of drugs and bioactive agents have been supported by a multitude of *in vitro* and preclinical studies, the development of these products through the R&D pipeline has not progressed at the same pace. All the lipid platforms discussed have entered clinical trials at various phases of development, with mostly encapsulation of local anesthetics, anti-infectives, or analgesics agents (Dubey et al., 2007; Elsayed et al., 2007; Nounou et al., 2008). Currently, there is only one lipid-based drug delivery platform for topical use on the market—estradiol topical emulsion (Estrasorb®) (Chiechi, 2004; Weissig et al., 2014). This product uses micellar nanoparticle technology to deliver estradiol to the blood circulation following topical application. In due course, we should expect to see a number of additional products for clinical use.

In order for the translational use of these carrier systems to the clinic, several issues still have to be addressed. Firstly, the safety of the different nano-delivery carriers following uptake needs to be explored further. Studies focused on the nanotoxicology of these delivery systems in human skin have been limited, especially with the newer classes, and is likely to vary according to the composition and size of the vesicles. For example, there is a need to evaluate the short-term and long-term effects of repeated applications of formulations containing relatively high concentrations of ethanol or surfactants on the skin. Secondly, structural and chemical stability during storage and following topical application would need to be further optimized to prevent premature release of the drug. Finally from a commercial development point of view, simplification of drug delivery design is required to allow efficient and reliable large-scale manufacturing for regulatory purposes. As the preparation of the newer classes of lipid vesicles involves similar methods to those used in the preparation of conventional liposomes, this has made the large-scale manufacturing and evaluation of such products less challenging. Although innovative and efficient, translation of these types of drug delivery systems to the clinic would need to show significant therapeutic advantage over existing therapeutic strategies, due to the added costs required in the manufacturing process.

### Conflict of interest statement

The author declares that the research was conducted in the absence of any commercial or financial relationships that could be construed as a potential conflict of interest.
